# Placenta-on-a-Chip as an In Vitro Approach to Evaluate the Physiological and Structural Characteristics of the Human Placental Barrier upon Drug Exposure: A Systematic Review

**DOI:** 10.3390/jcm12134315

**Published:** 2023-06-27

**Authors:** Femke A. Elzinga, Behrad Khalili, Daan J. Touw, Jelmer R. Prins, Peter Olinga, Henri G. D. Leuvenink, Harry van Goor, Sanne J. Gordijn, Anika Nagelkerke, Paola Mian

**Affiliations:** 1 Department of Clinical Pharmacy and Pharmacology, University Medical Center Groningen, University of Groningen, Hanzeplein 1, 9713 GZ Groningen, The Netherlands; f.a.elzinga@student.rug.nl (F.A.E.); b.khalili@student.rug.nl (B.K.); d.j.touw@umcg.nl (D.J.T.); 2 Department of Pharmaceutical Analysis, Groningen Research Institute of Pharmacy, University of Groningen, Antonius Deunsinglaan 1, 9713 AV Groningen, The Netherlands; a.p.nagelkerke@rug.nl; 3 Department of Obstetrics and Gynecology, University Medical Center Groningen, University of Groningen, Hanzeplein 1, 9713 GZ Groningen, The Netherlands; j.r.prins@umcg.nl (J.R.P.); s.j.gordijn@umcg.nl (S.J.G.); 4 Department of Pharmaceutical Technology and Biopharmacy, Groningen Research Institute of Pharmacy, University of Groningen, Antonius Deunsinglaan 1, 9713 AV Groningen, The Netherlands; p.olinga@rug.nl; 5 Department of Surgery, University Medical Center Groningen, University of Groningen, Hanzeplein 1, 9713 GZ Groningen, The Netherlands; h.g.d.leuvenink@umcg.nl; 6 Department of Pathology and Medical Biology, Pathology Section, University Medical Center Groningen, University of Groningen, Hanzeplein 1, 9713 GZ Groningen, The Netherlands; h.van.goor@umcg.nl

**Keywords:** placenta-on-a-chip, human placental barrier, microfluidic device

## Abstract

Quantification of fetal drug exposure remains challenging since sampling from the placenta or fetus during pregnancy is too invasive. Currently existing in vivo (e.g., cord blood sampling) and ex vivo (e.g., placenta perfusion) models have inherent limitations. A placenta-on-a-chip model is a promising alternative. A systematic search was performed in PubMed on 2 February 2023, and Embase on 14 March 2023. Studies were included where placenta-on-a-chip was used to investigate placental physiology, placenta in different obstetric conditions, and/or fetal exposure to maternally administered drugs. Seventeen articles were included that used comparable approaches but different microfluidic devices and/or different cultured maternal and fetal cell lines. Of these studies, four quantified glucose transfer, four studies evaluated drug transport, three studies investigated nanoparticles, one study analyzed bacterial infection and five studies investigated preeclampsia. It was demonstrated that placenta-on-a-chip has the capacity to recapitulate the key characteristics of the human placental barrier. We aimed to identify knowledge gaps and provide the first steps towards an overview of current protocols for developing a placenta-on-a-chip, that facilitates comparison of results from different studies. Although models differ, they offer a promising approach for in vitro human placental and fetal drug studies under healthy and pathological conditions.

## 1. Introduction

In 80% of pregnancies, at least one medicinal drug is used [1]. A recent study by EUROCAT in The Netherlands reported that 3% of this medication is classified to be potentially teratogenic and may therefore pose a risk for fetal development [1]. However, use of medicinal drugs during pregnancy is not always avoidable such as in patients with diabetes, epilepsy, or bipolar disorders. For most administered drugs, the risks for fetal development are unknown, with limited models available to predict the transport of xenobiotics across the human placental barrier which is a semipermeable layer separating the maternal and fetal circulation [2].

The placenta has a crucial role in fetal development via a variety of functions [3], including delivery of oxygen, glucose, and nutrients to the fetus and the removal of metabolic waste products and carbon dioxide from the fetus [4,5]. Furthermore, the placenta serves as a barrier to protect the fetus from maternal diseases and infections, as well as xenobiotics the mother may be exposed to [3,6]. The fulfillment of these functions by the placenta is essential for fetal growth and development during pregnancy [3]. The placental barrier consisting of a syncytiotrophoblast layer (cytotrophoblast cells) and endothelial cells separates the maternal and fetal circulations [3,7]. During pregnancy, the thickness of the trophoblast layers reduces to minimize the exchange distance and enhance the supply of oxygen and nutrients. Moreover, the villous vessels elongate and coil, thereby increasing their surface area and relative volume in the villous stroma. Furthermore, placental development involves the formation of chorionic villi, which contains a capillary network of placental tissue [7,8,9]. These chorionic villi undergo a maturation process that involves villous linear growth and branching, thereby increasing their surface area, enhancing exchange between mother and fetus [7]. Moreover, a variety of influx and efflux transporters are present at the placental interface to facilitate transport across the placental barrier and the expression of these transporters increases with the progression of pregnancy [6]. Because of the changing structure of the placental barrier during the progression of pregnancy it is challenging to study the placenta in regard to its barrier function.

Pregnant women are often excluded from clinical trials and post-marketing studies to avoid putting the fetus at risk. Therefore, different in vivo, ex vivo, and in vitro models were developed [4]. Animal models typically do not replicate the physiology of the human placenta due to interspecies differences in placentation, duration of pregnancy, and the changes that occur in the placental structure during pregnancy [10]. Ex vivo placenta perfusion models closely mimic the human placental physiology as genuine human placenta tissue is used [3]. Conversely, a limitation of this model is that explants of human placenta are difficult to maintain over extended periods of time and are therefore not ideal to evaluate long-term drug exposure [10,11]. Furthermore, the placental perfusion model is not suitable to study placental transfer during the most vulnerable period for the embryonic organogenesis: the first or early second trimester of pregnancy [12,13]. Non-chip in vitro models, such as Transwell systems, can be used to study drug transport across the placental barrier, placental hormone secretion, and invasion of trophoblasts [14,15]. Conversely, these models typically lack the physiological complexity of the human placenta in the form of a highly dynamic microenvironment. Therefore, new approaches are needed to overcome the challenges with current in vitro, ex vivo, and in vivo models.

Placenta-on-a-chip models are built around microfluidic chip devices, using cultured human placenta cells to analyze drug transport [2]. This technology does not require cell samples from the developing fetus, and thereby invasive procedures are avoided, making placenta-on-a-chip an ethically favorable approach [16]. These new platforms aim to mimic the structure and function of the human placental barrier as well as the blood circulation in placental tissue, which is a key advantage of these chip models [3,4]. The use of placenta-on-a-chip systems as an alternative to previously developed in vitro, in vivo, and ex vivo models has been demonstrated to have considerable potential for studying the fetal exposure of drugs administered to the mother [4]. Placenta-on-a-chip is not only suitable to analyze transport across the placental barrier under healthy conditions but additionally by expanding this model with, for instance, cell lines from placental tissue from women with preeclampsia, it is also possible to study placental transport under pathological conditions. This systematic review provides an overview of the currently available placenta-on-a-chip models and their applications. We aimed to identify knowledge gaps and provide the first steps towards an overview of current protocols for developing a placenta-on-a-chip, which makes comparison of results from different studies possible.

## 2. Materials and Methods

### 2.1. Search Strategy

This systematic review was conducted according to the PRISMA guidelines of 2020 [17] to retrieve studies on the placenta-on-a-chip model that investigate transport across the placental barrier. This study was not registered and therefore there is no registration number. A search was conducted using PubMed on 2 February 2023. In addition, Embase was used as a second database and a search was conducted on 14 March 2023. The search was performed with both the terms ‘placenta-on-a-chip’ and ‘placenta’. Table 1 lists the specific keywords for each term in PubMed. The search strategy from PubMed was converted to Embase, see Table 2.

### 2.2. Inclusion Criteria

This systemic review encompasses studies that explore the use of placenta-on-a-chip as a means to examine the placental barrier. As a result, the studies included had to focus on placenta-on-a-chip technology, a micro-engineered device that mimics the structure, function, and morphology of the human placenta. The type of cells used in the placenta-on-a-chip also needed to be an accurate representation of the cells present in the placental barrier interface. Additionally, papers addressing the transportation of substances across the placental barrier in the model were included. In addition, studies evaluating the integrity of the placental barrier under pathological conditions were included. No restrictions on the year of publication were placed on the search and only studies written in English were included. In addition, research included was limited to either experimental or observational, and reviews were excluded.

### 2.3. Study Selection

EndNote was utilized to eliminate duplicate articles. The title and abstract of the obtained articles were independently screened for relevance by two investigators (FE and BK). Subsequently, the full texts of studies with relevance to this study were read. Studies meeting the inclusion criteria were included in this systematic review. In case of disagreement between the two researchers, a third investigator (PM) was consulted.

### 2.4. Data Extraction

Data were extracted from the included studies. One author (FA) was responsible for the data extraction of half of the included articles, whereas a second author (BK) performed extraction of the other half of the studies. Thereafter, both investigators (FE and BK) checked the extracted data of each other. For the systematic approach of this review paper, all extracted data of each eligible article were included in a data table using Microsoft Word by one author (FE). The data table was checked by a second author (BK) and subsequently by a third investigator (PM). The following data were extracted from the eligible articles: type of testing, aim of the study, fabrication method of the microfluid chip, and main findings of each study. In addition, compound properties such as concentration, size, and exposure time were extracted. If reported, the properties of the fabricated placental barrier that were collected included the flow rate, pore size, and shear stress.

## 3. Results

### 3.1. Study Selection and Data Extraction

**Figure 1:**
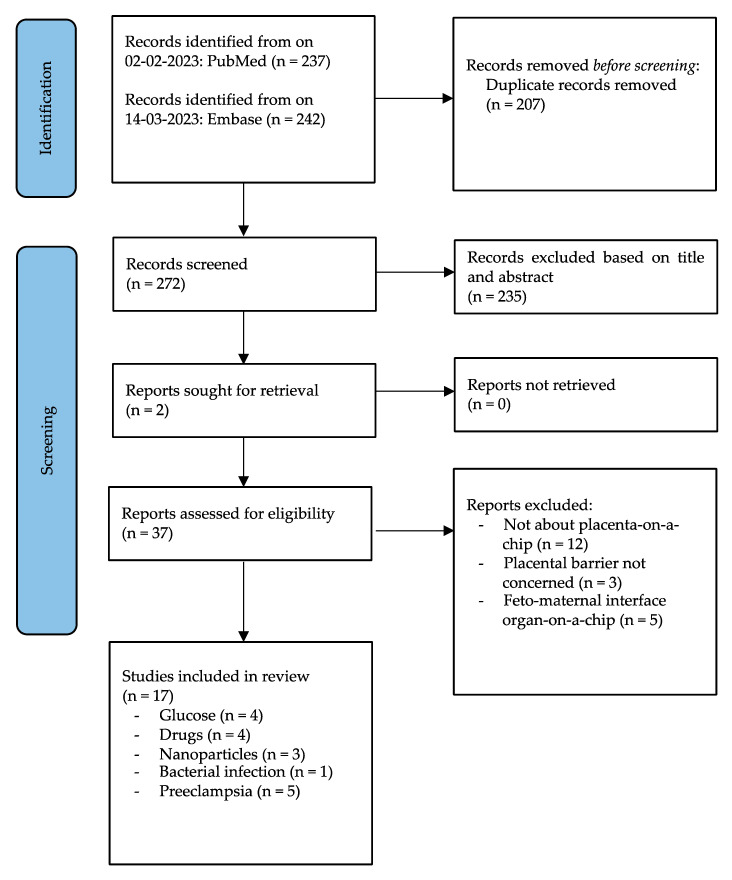
PRISMA flow diagram.

For each eligible article study, selection and data extraction were performed. From PubMed, 237 articles were obtained. Additionally, 242 articles were obtained by Embase, which was used as a second database. A total of 207 duplicate articles were removed using EndNote (version 20). After deduplication, 272 articles were screened based on title and abstract after which 235 studies were excluded. Subsequently, the text of 37 articles was read in full. After reading the full papers, 17 articles were included whereas 20 articles which did not meet the inclusion criteria were excluded, see Figure 1.

From these 20 articles, 12 did not study placenta-on-a-chip (60%). In three articles, the placental barrier was not subjected to study (15%). Moreover, five articles studied the feto–maternal interface in an organ-on-a-chip system (25%) instead of the placenta. The feto–maternal interface is highly specialized tissue which includes both the decidua and the placenta. However, in this systematic review we only focus on the placenta parenchyma and therefore studies studying the feto–maternal interface were excluded.

### 3.2. Manufacture of Placenta-on-a-Chip Microfluidic Device

**Figure 2:**
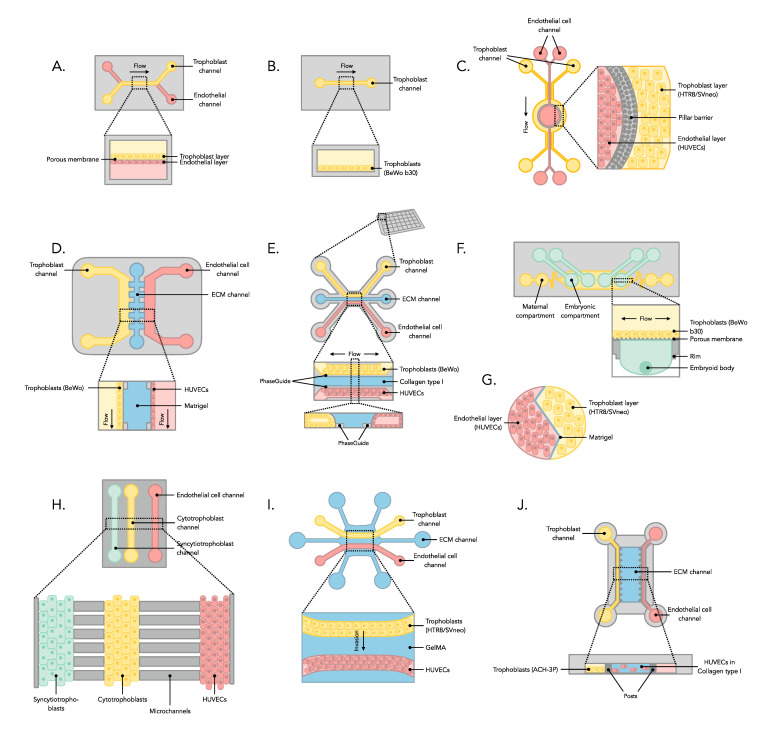
Overview of the different designs for placenta-on-a-chip devices discussed in this systematic review. (**A**) Two-channel placental barrier chips, in which placental trophoblasts (BeWo (b30) or JEG-3) are cultured in one of the channels and endothelial cells (HPVECs and HUVECs) in the other, separated with a porous membrane. The porous membrane is typically coated with a thin layer of ECM (Collagen type I, Fibronectin or ECL). Used in [2,18,19,20,21,22]. (**B**) The iBidi μ-Slide I^0.4^ seeded with trophoblast cells (BeWo b30), as used in [10]. (**C**) The SynVivo 3D microfluidic chip used in [31]. The trophoblasts used (HTR8/SVneo) are separated from the endothelial cells (HUVECs) by a layer of PDMS pillars. Spacing between the pillars is 3 μm, allowing molecules to diffuse and cells to migrate through. (**D**) PDMS-based microdevice with three channels in parallel as described in [24]. The two flanking channels are used for cells (trophoblasts in the form of BeWo and endothelial cells in the form of HUVECs). The central channel is used for ECM (Matrigel). (**E**) The 3-lane Organoplate, seeded with trophoblasts (BeWo in the studies reported), ECM (Collagen type I or a mixture of Collagen type I and IV), and endothelial cells (HUVECs) [4,29]. (**F**) Microdevice allowing culture of trophoblasts (BeWo b30) on top of a porous membrane [25]. Underneath, embryoid bodies are formed and cultured in hanging drops. (**G**) PDMS-based interdigitated platform for studying interaction between trophoblasts (HTR8/SVneo) and endothelial cells (HUVECs), separated by a thin layer of ECM (Matrigel) [28]. (**H**) The PLA-OOC device reported in [23]. Three parallel channels support (syn)cytotrophoblasts (BeWo) and endothelial cells (HUVECs). The channels are connected by smaller microchannels. (**I**) Gel patterning microfluidic chip [27]. A cell culture channel for trophoblasts (HTR8/SVneo) is separated from an endothelial cell channel (HUVECs) by a GelMA structure. (**J**) The AIM Biotech idenTx chip [30]. A central ECM compartment is used for the culture of endothelial cells (HUVECs) in ECM (Collagen type I). This is separated from the trophoblast channel (ACH-3P cells) by microposts. The endothelial cell channel supplies endothelial cell medium. Systems A-D use externally applied flow. Systems E-F rely on gravity (tilting back and forth) to establish flow. System G-I does not incorporate flow and system J applies volume differences to establish interstitial flow. Please note, drawings are not to scale.

This systematic review included 17 studies that used placenta-on-a-chip to investigate the physiology of the human placenta in vitro under healthy and pathological conditions. Among the included studies (see Table 3, Table 4, Table 5, Table 6 and Table 7), 12 studies used soft lithography for the in-house fabrication of the microdevice, relying on polydimethylsiloxane (PDMS) [2,18,19,20,21,22,23,24,25,26,27,28]. Two studies included made use of the commercially available 3-lane OrganoPlate from Mimetas [4,29], one used the AIM Biotech idenTx chip [30], one the SynVivo 3D microfluidic chip [31], and one the iBidi μ-Slide I^0.4^ [10]. The majority of studies employed microdevices composed of a multilayered structure consisting of a maternal channel and a fetal channel, separated by a thin semipermeable membrane. Second, a chip design with parallel lanes, separated by an extracellular matrix (ECM), to represent the material and the fetal side was frequently used. A schematic overview of the systems is shown in Figure 2.

All models rely on the culture of human trophoblast cell lines inside the devices. These cells are cultured in one of the channels of the chip (see Figure 2). The trophoblast cells used in 12 of the included studies were the BeWo or BeWo b30 cells [2,4,10,18,21,22,23,24,25,26,29]. Three studies used HTR8/SVneo cells [27,28,31], one study ACH-3P cells [30], and one study JEG-3 cells [20].

Other channels in the microfluidic devices were typically used for culturing human endothelial cells (see Figure 2). It was reported that 14 studies used human umbilical vein endothelial cells (HUVECs) [4,10,19,20,21,22,23,24,26,27,28,29,30,31] and two studies human placental vascular endothelial cells (HPVECs) [2,18]. Furthermore, one study included embryoid bodies in combination with BeWo b30 cells to recapitulate exposure of the early embryo [25].

The porous membrane, used in the majority of included studies, was placed between the maternal compartment and the fetal compartment. This membrane was coated with ECM to enable growth and maturation of the trophoblast and endothelial cell lines before and after perfusion. Secondly, in systems using the parallel channel design, a thicker layer of ECM was used as a stromal barrier. Different ECMs were used among the included studies (see Table 3, Table 4, Table 5, Table 6 and Table 7). Seven studies used collagen type I [4,19,20,23,25,26,30], six studies used fibronectin [2,18,20,27,28,31], two studies used gelatin [10,20], two studies used a combination of entactin, collagen IV and laminin [21,22], two studies used Matrigel [24,28], one study used chitosan [24], one study used methacrylated gelatin (GelMA) [27], and one study used a combination of collagen type I and IV [29]. Normally, in the human placenta, ECM consists of a mixture of laminin, collagen IV, fibronectin, glycoproteins, and growth factors, including vascular endothelial growth factor (VEGF) which is crucial for placental growth [32,33,34]. By connecting a perfusion system to the microchannels, the dynamic environment of the placenta was mimicked in the majority of studies. This is crucial to replicate the physiological and functional conditions of the placenta in vivo [4,20]. Therefore, the pore size of the porous membrane, the ECM used, the flow rate, and generated shear stress are essential factors to replicate the human placental environment (see Table 3, Table 4, Table 5, Table 6 and Table 7). Nine studies reported on the flow rate [10,18,19,20,21,22,24,25,31] ranging from 0.6 to 1373.4 μL/h. The pore size of the porous membrane was reported by seven studies [2,18,19,21,22,25,26] and was either 0.4, 1, or 3 μm. One study explicitly mentioned pore size of the ECM used, namely 2–3 μm [4]. Five studies reported the shear stress generated in the microfluidic device [4,10,24,25,31], ranging from 0.0003 to 1.41 dyne/cm^2^. Furthermore, the properties of compounds (see Table 3, Table 4, Table 5, Table 6 and Table 7), such as drugs, nanoparticles, and nutrients, are important for investigating the effect on the permeability placental barrier. The concentration was reported by 16 studies [2,4,10,18,19,20,21,22,23,24,25,27,28,29,30,31]. Though not relevant for all included studies, the particle size was reported by three studies [10,24,25]. The time exposed to a specific particle was reported in 17 studies [2,4,10,18,19,20,21,22,23,24,25,26,27,28,29,30,31].

In summary, placenta-on-a-chip systems in general are microfluidic devices consisting of a maternal and a fetal channel which are either separated by a porous membrane coated with ECM or by a gel layer of ECM. Different trophoblast and endothelial cell lines were used among the included studies. In addition, differences in flow rate and shear stress are substantial between included studies. Moreover, generation of a dynamic environment in an in vitro placenta, which is partly determined by properties of the placental barrier, is important to mimic the human placenta in vivo.

### 3.3. Analysis of Experiments from Placenta-on-a-Chip

Placenta-on-a-chip is used to study a broad range of applications. This includes glucose transfer and drug transport across the placental barrier. Placenta-on-a-chip is also used to investigate the interactions of nanoparticles with the placental barrier. Furthermore, this microfluidic chip model is not only used to study placental physiology under healthy conditions but also under pathological conditions including bacterial *E. coli* infection and preeclampsia. The paragraphs below present an overview of the applications for which placenta-on-a-chip is used to study the key characteristics of the human placental barrier under healthy and pathological conditions.

#### 3.3.1. Glucose

A total of four studies on glucose transfer across the placental barrier were included [4,18,19,20]. Table 3 provides an overview regarding the characteristics of all four studies. Among these studies, three [18,19,20] evaluated the rate of glucose transfer, and one study investigated glucose transfer in the presence of placental malaria [4].

The study by Blundell et al. [18] determined that in a placenta-on-a-chip co-culture model of trophoblasts and endothelial cells, the percent rate of placental glucose transfer was 34.8%. This corresponds with glucose transfer rates in ex vivo models, which range from 26.5 to 38.3%. However, in a static Transwell co-culture model of trophoblasts and endothelial cells the percent rate of placental glucose transfer was 22.5%. This value is not in the range of glucose rate obtained in perfused ex vivo human placenta. This suggests that placenta-on-a-chip more capable of recapitulating glucose transport rates from maternal to fetal compartments than Transwell in vitro models. Glucose transport percent rates in another study [20] using HUVEC monoculture placenta-on-a-chip models found values ranging from 45.5 to 93%. However, in a co-culture model of JEG-3 cells and HUVECs, glucose rates ranged from 17.3 to 39.1%. This validates the role of trophoblast cells in the function of the placenta mediating glucose transport to provide adequate amounts of glucose to the fetus, thereby preventing excessive glucose supply resulting in adverse pregnancy outcomes. Additionally, these findings were supported by the study of Mosavati et al. [19], which demonstrated that the rate of glucose transfer was reduced in the co-culture microfluidic model compared with monoculture models (35% vs. 66.5%).

Another study by the same group [4] examined the sequestration of infected erythrocytes (IEs) via cytoadhesion to chondroitin sulfate A (CSA), expressed on the surface of trophoblast cells and a key feature of placental malaria. The authors reported that these IEs impaired glucose transfer across the placental barrier compared with control groups (difference 113.1%).

Together, these findings demonstrate that placenta-on-a-chip is a useful platform for glucose transfer investigations across the placental barrier as glucose is the primary source of energy for the development of the fetus. The relevance of placenta-on-a-chip was demonstrated over Transwell models. In addition, the role of trophoblasts was validated in the functioning of the placental barrier. Moreover, glucose transfer between the maternal and fetal compartment was impaired by malaria.

#### 3.3.2. Drugs

In total, four studies investigating placental drug transfer were included in this review (Table 4) [2,21,22,23]. In order to test drug transport across the placental barrier, the drug was introduced to the maternal compartment and subsequently the concentration was measured in the fetal compartment at different time points.

One of the studies [21] assessed the transport of Naltrexone/6ß-Naltrexol, used to treat opioid addiction, across the placental barrier. The authors reported that in an acellular model 10.3–10.5% of the initially introduced maternal concentration was measured in the fetal compartment. However, in a co-cultured model of BeWo cells and HUVECs, the mean concentration in the fetal compartment was only 2.2–2.5% compared with the initial concentration in the maternal compartment. Furthermore, after six hours the mean concentration of Naltrexone/6ß-Naltrexol in the co-cultured model began to rise in comparison with measured mean concentrations between one and six hours until the end of the experiment, which indicates disruption of the placental barrier.

A subsequent study of the same group [22] investigated the rate of placental caffeine transport by identifying the time required to reach steady state concentrations in both the maternal and fetal compartment. Identifying these concentrations helps to determine the safe amount of caffeine which can be taken by the mother without reaching toxic levels on the fetal side. Introduction of a caffeine concentration of 0.25 mg/mL to the maternal compartment resulted in a steady state concentration of 0.0033 mg/mL in the fetal compartment after five hours. In addition, a steady state concentration of 0.1513 mg/mL was reached in the maternal compartment after 6.5 h.

Blundell et al. [2] evaluated glyburide transport over the placental barrier. Glyburide is a medicinal drug that is prescribed to pregnant women diagnosed with gestational diabetes. The use of this drug by the mother is necessary to maintain normal glucose levels during pregnancy; however, exposure to the fetus may be harmful. A decrease in glyburide concentration (five-fold reduction) was reported in the maternal compartment of the co-culture model of BeWo cells and HPVECs after 30 min, implying that a significant amount transferred to the fetal compartment. Conversely, after three hours of glyburide perfusion, the concentration in the maternal compartment began to rise again, reaching a concentration that was four times higher compared with the initially introduced glyburide concentration. These results suggested that an active efflux transport system of the placental barrier which is thought to be mediated by breast cancer resistance protein (BCRP) impaired fetal exposure to glyburide by transporting the drug from the fetal compartment to the maternal compartment.

Another study [23] demonstrated that the microfluidic models are not only able to recapitulate placental transport of Rovustatin and Pravastatin, but also that the cellular layers of the placental barrier are capable of metabolizing these drugs. The authors reported that Pravastatin was metabolized within 24 h by all cell layers by glucuronidation. Rovustatin was metabolized within 8 and 24 h by the trophoblast cell layer involving CYP3A4 and CYP2D6. Both Pravastatin and Rosuvastatin are potential drugs to decrease inflammation associated with preeclampsia. Therefore, it was determined that under the influence of Pravastatin, both pro-inflammatory (IL-6) and anti-inflammatory cytokines (IL-4 and IL-10) were produced. Rosuvastatin produced only anti-inflammatory cytokines (IL-4) and may therefore be a more effective drug for treating inflammation associated with preeclampsia.

The findings of these four studies together reveal that placenta-on-a-chip can be an approach to study transport of Naltrexone/6ß-Naltrexol, caffeine, glyburide, and statins across the placental barrier. Moreover, these microchip devices are able to replicate the active efflux system of the ex vivo human placenta as well as metabolizing maternally administered statins by the different cell layers in the placental barrier.

#### 3.3.3. Nanoparticles

In total, three studies on placental nanoparticle exposure were included, of which the key characteristics are provided in Table 5 [10,24,25]. Among these studies, two studies [24,25] investigated the effect of a specific nanoparticle on the placental barrier integrity and one study [10] evaluated the uptake of nanoparticles by trophoblast cells.

The first study [24] investigated the impact of titanium dioxide nanoparticles (TiO_2_ NPs) on the placental barrier. The authors demonstrated that exposure to the highest concentration (200 μg/mL) of TiO_2_ NPs stimulated generation of reactive oxygen species (ROS), considerably causing trophoblast cell death, damaging the placental barrier. It was reported that with both low and high (50 and 200 μg/mL) concentrations of TiO_2_ NPs, the permeability of the placental barrier was disrupted. Moreover, upon exposure to TiO_2_ NPs, trophoblast cells attracted maternal macrophages, which was associated with impaired placental barrier function.

Another study [25] investigated direct and indirect early embryotoxicity upon exposure to carboxyl-modified polystyrene microparticles (PS-MPs). In this study, embryoid bodies were cultured in the fetal compartment to mimic early embryonic development. It was reported that after three days of exposure to PS-MPs, concentrations varied from low to high (1, 10, and 100 μg/mL), no significant translocation over the trophoblast barrier was observed, which indicated that there were no direct embryotoxic effects. The reported value for intracellular ATP content of embryoid bodies as markers for viability and proliferative capacity was reduced and declined to 0.84, 0.78, and 0.84-fold after exposure to 1, 10, and 100 μg/mL PS-MP, respectively. Because there was no translocation of PS-MPs and reduced ATP content in embryoid bodies, the authors expected that secondary placental reactions were responsible for indirect embryotoxic effects.

Abostait et al. [10] investigated the extent of cellular uptake of chondroitin sulphate A (CSA)-conjugated PEGylated liposomal nanocarriers by different degrees of trophoblast syncytialization as well as in dynamic conditions using the iBidi μ-Slide I^0.4^. It was reported that the production of ß-hCG, indicating trophoblast syncytialization, was increased under dynamic conditions compared with static conditions, with a difference of 166.7%. According to the results, forskolin treatment increased the degree of syncytialization further. Upon prolonged exposure to forskolin (48 and 72 h), this increase was significant, with a difference of 96.2% compared with dynamic control. Moreover, a combination of both the dynamic environment and the extent of syncytialization can increase the uptake of CSA-conjugated PEGylated liposomes by trophoblast cells (difference 29.2%).

Together, the results of these studies showed that prolonged exposure to these nanoparticles caused disruption of the placental barrier which was accompanied by the production of reactive oxygen species leading to trophoblast cell death. In addition, it was reported that nanoparticle exposure can activate the innate maternal immune system. Furthermore, recapitulating the dynamic microenvironment was shown to be crucial to maintain the integrity of the placental barrier.

#### 3.3.4. Bacterial Exposure

Table 6 provides the key characteristics of the included study under pathological conditions of bacterial infection. The study [26] fabricated a placenta-on-a-chip system to analyze placental inflammatory responses to bacterial *E. coli* infection in vitro. According to the findings, after placental cells were inoculated for six hours with *E. coli* bacteria, trophoblast cells produced larger amounts of inflammatory cytokines including IL-1α, IL-1ß, IL-6, and IL-8. Additionally, after inoculation of trophoblasts and endothelial cells with *E. coli*, macrophages (THP-1) were introduced onto the trophoblast layer of the chip. It was demonstrated that more macrophages were attached to the epithelium, indicating activation of the maternal innate immune system in the *E. coli*-infected human placenta, causing acute placental inflammation associated with loss of placental function, which might result in abnormal fetal development and preterm birth.

#### 3.3.5. Preeclampsia

Five studies on preeclampsia were included, Table 7 provides the key characteristics [27,28,29,30,31]. Among these studies, one study [30] investigated the inflammatory signaling and vascular network formation in preeclampsia, three studies [27,28,31] assessed trophoblast migration and invasion under different oxygen conditions, and one study [29] investigated placental damage causing preeclampsia.

The first included study [30] established a placenta-on-a-chip model to evaluate the expression of FK506-binding protein-like (FKBPL) and galectin-3 (Gal-3) associated with vascular dysfunction in preeclampsia. The authors found that in human plasma and placental tissue from women with preeclampsia both circulating, placental FKBPL (difference in FKBPL levels between preeclampsia vs. normotensive pregnancy: plasma 60.2% and placental 128.0%) and Gal-3 proteins (difference in Gal-3 protein levels between preeclampsia vs. normotensive pregnancy: plasma 30.0% and placental 197.6%) were increased compared with women with a normotensive pregnancy. Placenta-on-a-chip was used to determine FKBPL and Gal-3 protein signaling associated with inflammatory conditions in preeclampsia. In order to mimic the conditions in preeclampsia, trophoblasts and endothelial cells were treated with TNF-α; a pro-inflammatory cytokine that is highly expressed on/by endothelial cells in preeclampsia. It was demonstrated that in co-culture microfluidic model both FKBPL and Gal-3 signaling were significantly increased after treatment of trophoblasts and endothelial cells with TNF-α (10 ng/mL) for 24 h. When focusing on placental vascular network formation, it was demonstrated in the placental microfluidic model that placental vascular network formation was impaired upon exposure to TNF-α for 72 h. Furthermore, it was reported that trophoblasts were responsible for reduced placental vascular network formation and that the migration of trophoblasts was stimulated by endothelial cells.

A second study looking at trophoblast invasion [31] reported that folic acid stimulated trophoblast invasion by modulating MMP-2 expression in a co-culture model of HTR8/SVneo cells and HUVECs. No quantitative information on this study was reported by the authors [31]. Additionally, trophoblast invasion was further enhanced in the absence of endothelial cells. This contradicts the result reported by the first included study described above [30]. Differences between these studies are (i) the trophoblast cell line used, (ii) the set-up of the microdevice in terms of flow rate, and (iii) the ECM and barrier structure used.

The third study [28] developed a PDMS-based interdigitated chip device to analyze trophoblast invasion and migration towards HUVECs in hypoxic (0.5% O_2_) and normoxic (21% O_2_) conditions. A hypoxic environment is essential for correct development of the placenta. This involves stimulation of trophoblast invasion. Insufficiency in trophoblast invasion at this stage, together with inadequate spinal artery remodeling, may lead to placental hypoxia and preeclampsia at later stages. The authors reported that hypoxic conditions induced MMP-9 expression in trophoblast cells by 0.6-fold at 12 h and 3.7-fold at 36 h. MMP-9 expression is associated with ECM degradation and trophoblast invasion. As such, trophoblast migration was promoted under hypoxic conditions and even further increased with longer exposure times (24 and 36 h) with invasion distances ranging from ±1500 μm at 12 h to ±2000 μm at 36 h. In addition, the fourth study included [27], reported the promotion of trophoblast migration towards HUVECs in a hypoxic environment (3% O_2_). The authors developed a microdevice in which trophoblast cells were enabled to migrate through an ECM composed of methacrylated gelatin. The mean invasive distance increased from ±410 μm in a normoxic environment (21% O_2_) to ±670 μm in the hypoxic environment.

The last study [29] investigated damage to the placental barrier associated with preeclampsia by exposing cultured cells to different oxygen levels and application of different perfusion tensions (medium/static perfusion) in the microfluidic model. It was reported that after 24 h under normoxic conditions (20% O_2_ with medium perfusion) as well as hypoxic conditions (1% O_2_ with medium perfusion) no changes in the syncytium permeability were observed, with 1.23 and 1.29-fold reduction in transepithelial electrical resistance (TEER) of the trophoblast barrier over time. Conversely, under ischemic conditions (1% O_2_ with static medium conditions), significant damage to the syncytium was observed, with a TEER value of −1.9.

Therefore, a placenta-on-a-chip microfluidic device can be used to investigate inflammatory signaling, vascular network formation, and barrier damage as well as trophoblast migration/invasion with different oxygen concentrations, for example, for studying preeclampsia.

## 4. Discussion

The use of drugs during pregnancy has increased significantly over the past years [35]. Quantification of placenta drugs transfer and potential risks on fetal development after exposure to maternally administered drugs are largely unknown. Therefore, a novel strategy is required to experimentally determine fetal drug exposure, especially in the first trimester of pregnancy, as during this period the embryo is most vulnerable to teratogenesis as organogenesis takes place [1,13].

This systematic review highlights the potential benefits of utilizing placenta-on-a-chip technology for better comprehension of placental biology, and its effect on transfer of drugs and other compounds. The included studies have shown that placenta-on-a-chip models can be used for a wide range of applications such as glucose transfer [4,18,19,20], transfer of various drugs [2,21,22,23], gain insight into mechanics of placental growth [27,29,30], mimicking active efflux transporter function of the placental barrier [2], and to study the impact of nanoparticles on the placental barrier [10,24,25]. Furthermore, it was demonstrated that placenta-on-a-chip has the capability to recapitulate the placental barrier under pathological conditions including bacterial infections [26], placental malaria [4], and preeclampsia [27,28,29,30,31].

Placenta-on-a-chip technology is a promising platform to investigate the physiology of the human placenta, as it is able to simulate blood flow and provides a more accurate representation of the dynamic microenvironment within the placenta compared with other in vitro placental models [3,4,20]. Furthermore, placenta-on-a-chip offers a more ethical approach compared with in vivo and ex vivo models since it does not require cell samples of the developing fetus, and thereby no invasive procedures are required to obtain fetal cells avoiding putting mother and fetus at risk [4,16]. Recently, protocols have also been established with which trophoblast organoids can be cultured [36,37,38,39]. Though this requires an initial source of primary human material, it does offer the opportunity to incorporate in placenta-on-a-chip systems in the future because the reliance on trophoblast cell lines in such systems can be eliminated. We believe this will further advance the physiological relevance of placenta-on-a-chip models but will also improve our ability to assess biological variation within the human population. With the ultimate goal of improving the health outcomes for both mother and fetus, this technology represents a crucial tool in responding to the urgent need for more sophisticated experimental models to explore fetal exposure and development.

There are other organ-on-a-chip models which are already further developed. Liver-on-a-chip models can simulate liver functions such as metabolism, detoxification, and bile acid secretion [40]. Liver-on-a-chip technology is able to predict metabolism of drugs and can identify potential toxic drug effects before it is tested on humans [40], and therefore the prospective is that this platform will be the main method in preclinical drug testing [41]. Additionally, other organ-on-a-chip models are in development, resembling the spleen, bone marrow, and lymph nodes, to gain more insight on the immune system [42]. Moreover, the development of lung-on-a-chip offers a platform for better understanding of the underlying mechanisms of pathophysiological conditions within the lungs including lung cancer, asthma, COPD, and pulmonary fibrosis [43,44,45,46,47]. Intestine-on-a-chip can recapitulate normal intestinal functions including nutrient and drug absorption, mucus secretion by intestinal epithelium, digestive capacity of gastrointestinal enzymes, and the microbial flora present in the gastrointestinal tract [48,49]. Therefore, organ-on-a-chip microfluidic technology can recapitulate the physiological characteristics of different human organs under healthy and pathological conditions. Therefore, more comprehensive research on placenta-on-a-chip technology may lead towards a standardized protocol, which makes it possible to expand the range of applications for which placenta-on-a-chip can be used.

With this systematic review, we made the first step towards an overview of current protocols, which makes it easier to compare the results obtained from different studies. There are a number of components involved in the currently available placenta-on-a-chip approaches that are important to include in an overview of current protocols, and these are discussed below.

Many studies used BeWo (b30), ACH-3P, or JEG-3 cell lines in the maternal channel, which have been shown to be successful in simulating the characteristics of the trophoblastic epithelium of the placental barrier [2,4,10,18,19,20,21,22,23,24,25,26,29,30]. However, these cultured placental cells are derived from choriocarcinoma cell lines [18,30,50]. It is unknown if these cancer-derived cells have the capacity in the fabricated microfluidic systems to represent the normal epithelium of the placenta [18]. Therefore, as mentioned above, future studies can incorporate primary human trophoblast cells to address this limitation.

Furthermore, most studies reported the use of PDMS as porous membrane in their fabricated placenta-on-a-chip devices. However, PDMS is a polymer which is able to absorb small hydrophobic drug compounds leading to drug loss in the microfluidic device [51]. This problem was also reported in the study of Blundell et al. [2] as PDMS has the capacity to absorb antidiabetic drug glyburide leading to drug loss in the maternal compartment. Recent studies have shown that a lipophilic coating of the PDMS surface reduces drug absorption [51]. Additionally, alternative materials for the fabrication of microchip devices are being sourced. The study of Ghorbanpour et al. [30] reported the use of a commercial chip system based on cyclic olefin polymers (COPs). This seems to be a suitable alternative material for use in placenta-on-a-chip devices [52]. Therefore, using lipophilic coatings to prevent drug absorption of PDMS or materials such as COP may represent attractive strategies to prevent undesired interactions of small molecules with the microdevice.

It is challenging to replicate the dynamic environment of the human placenta in an in vitro model. The study of Lee et al. [20] reported that the generated shear stress in their placenta-on-a-chip device was considerably lower compared with those within the human placenta under healthy conditions. Therefore, it may be useful to translate the shear stress conditions of in vivo placental perfusion models to placenta-on-a-chip technology. However, among recent studies using placental perfusion models to investigate human placental physiology, there is a wide variety in the reported conditions for shear stress. In general, the shear stress conditions are low if the shear stress is smaller than 2 dyne/cm^2^ preventing migration of trophoblast. Conversely, shear stress conditions are increased if the generated shear stress is between 4 and 6 dyne/cm^2^, leading to trophoblast migration [53]. Maintaining dynamic conditions within the microfluidic model is crucial to replicate the essential physiological and structural characteristics of the human placenta such as hormone production and transporter function. Therefore, further studies should focus on generating dynamic conditions with more appropriate shear stresses to simulate more accurately the microenvironment within the placenta.

Even though there is room for improvement on the current available placenta-on-a-chip models, this systematic review points us in the right direction by providing an overview of current protocols. This recently developed approach holds great promise in investigating physiological and structural characteristics of the human placenta. However, the use of placenta-on-a-chip is a relatively new field of research and other organ-on-a-chip models are already further developed. In order to use placenta-on-a-chip to investigate a broader range of applications, for instance placental drug metabolism or immune cell behavior of the placental barrier, further development of this platform is required. Eventually the perspective is that placenta-on-a-chip will become the new gold standard for investigating the human placenta in healthy and pathological conditions; therefore, further investigations are required to create an overview of current protocols. The future prospective is that further development will make placenta-on-a-chip a novel technology towards replicating placental dynamics during the different phases of pregnancies.

This systematic review shows that placenta-on-a-chip is an innovative novel experimental strategy that is capable of mimicking the physiological and structural aspects of the human placenta. This newly developed technology offers a more ethical approach and provides a more accurate representation of the dynamic microenvironment of the human placenta compared with other methods. However, more knowledge is required to improve the components of this new field of research. With this review, we summarized the available knowledge and provided first steps towards an overview of the current protocols. Although placenta-on-a-chip is in its infancy, the prospective is that this platform has the potential to become the new gold standard for investigating both fetal drug exposure as well as the physiology and structure of the human placental barrier under different conditions.

## Figures and Tables

**Table 1:** Key terms used in search strategy in PubMed.

Placenta-on-a-Chip	Placenta
“Placenta-on-a-chip”[Mesh] placenta-on-chip*[tiab], placenta model*[tiab], placental model*[tiab], placenta in vitro model*[tiab], “Lab-On-a-chip devices”[Mesh] on-a-chip*[tiab], nanochip*[tiab], microchip*[tiab], microfluidic*[tiab]	“Placenta”[Mesh] placenta*[tiab], placentome*[tiab], decidua*[tiab], deciduoma*[tiab], trophoblast*[tiab], cytotrophoblast*[tiab], syncytiotrophoblast*[tiab]

**Table 2:** Key terms used in search strategy in Embase.

Placenta-on-a-Chip	Placenta
‘placenta-on-a-chip’/exp placenta-on-chip*:ab,ti, placenta model*:ab,ti, placental model*:ab,ti, placenta in vitro model*:ab,ti, ‘lab on a chip’/exp on-a-chip*:ab,ti, nanochip*:ab,ti, microchip*:ab,ti, microfluidic*:ab,ti	‘placenta’/exp placenta*:ab,ti, placentome*:ab,ti, decidua*:ab,ti, deciduoma*:ab,ti, trophoblast*:ab,ti, cytotrophoblast*:ab,ti, syncytiotrophoblast*:ab,ti

**Table 3:** Key characteristics of included studies on glucose.

Type of Testing	Aim	Cultured Cells	Properties	Chip Type	Main Findings	Reference
Glucose	Placental glucose transport	BeWo b30 cells (Trophoblast cell line) HPVECs (Endothelial cell line)	Substance tested: Glucose concentration: Maternal channel: 10 mM Fetal channel: 5.5 mM Exposure 2 h Placental barrier: Flow rate: 100 μL/h Porous membrane: polycarbonate, pore size 1 μm ECM: Fibronectin coating (0.1 mg/mL)	In-house manufactured using soft lithography, PDMS-based	- Shear stress stimulated the formation of microvilli by trophoblasts. - Placenta-on-a-chip co-culture model: %rate of glucose transfer is 34.8%. - Transwell co-culture model: %rate of glucose transfer is 22.5%.	Blundell et al. [18]
Glucose	Placental glucose transport	JEG-3 (Trophoblast cell line) HUVECs (Endothelial cell line)	Substance tested: Glucose concentration: Maternal channel: 25 mM Fetal channel: 6.1 mM Exposure 68 h Placental barrier: Flow rate: 30 μL/h Porous membrane: Vitrified Collagen-I (2.43 mg/mL) ECM: Fibronectin coating (40 mg/mL, upper channel) and gelatin coating (1.5%, lower channel)	In-house manufactured using soft lithography, PDMS-based	- Co-culture model: smallest amount of glucose transferred from maternal to fetal compartment. - HUVECs monoculture model: largest amount of glucose transferred from maternal to fetal compartment. - JEG-3 monoculture model: largest reduction in glucose concentration to co-culture, HUVECs monoculture acellular models. - Glucose permeability coefficients indicate that endothelial cells were more glucose permeable than trophoblasts.	Lee et al. [20]
Glucose	Placental glucose transport	BeWo cells (Trophoblast cell line) HUVECs (Endothelial cell line)	Substance tested: Glucose concentration: Maternal channel: 7.2 mM Fetal channel: 5.6 mM Exposure 2 h Placental barrier: Flow rate: 50 μL/h Porous membrane: polycarbonate, pore size 0.4 μm ECM: Collagen type I coating (concentration n.r.)	In-house manufactured using soft lithography, PDMS-based	- Smaller glucose diffusion rate in model with co-cultured cells compared to models with monoculture and microdevice with no cells. - Glucose diffusion rate increases with membrane porosity (numerical simulation). - Glucose diffusion rate decreases with flow rate (numerical simulation)	Mosavati et al. [19]
Glucose	Placental glucose transport under influence of placental malaria	BeWo cells (Trophoblast cell line) HUVECs (Endothelial cell line)	Substance tested: Glucose concentration: Maternal channel: 8.9 mM Fetal channel: 5.4 mM Exposure 2 h Placental barrier: Flow rate: n/a. Shear stress: 0–1.41 dyne/cm^2^ ECM: Collagen type I gel (0.4 mg/mL), pore size 2–3 μm	3-lane OrganoPlate (MIMETAS)	- Glucose consumption by infected erythrocytes is two orders of magnitude smaller compared to BeWo cells and HUVECs. - In CSA-binding erythrocytes infected with CS2 parasites the amount of glucose transport across the placental barrier was much lower compared to non-infected erythrocytes.	Mosavati et al. [4]

**Table 4:** Key characteristics of included studies on drugs.

Type of Testing	Aim	Cultured Cells	Properties	Chip Type	Main Findings	Reference
Naltrexone/ 6ß-Naltrexol	Placental drug transport and fetal drug exposure	BeWo cells (Trophoblast cell line) HUVECs (Endothelial cell line)	Substance tested: Naltrexone concentration: 293 mM 6ß-Naltrexol concentration: 291 mM Exposure 8 h Placental barrier: Flow rate: 50 μL/h Porous membrane: polyethylene terephthalate, pore size: 0.4 μm ECM: Entactin collagen IV-laminin coating (10 μg/mL)	In-house manufactured using soft lithography, PDMS-based	- Shear stress stimulated the formation of microvilli on maternal compartment. - Acellular device: 10.5% and 10.3% of initial maternal concentration NTX and 6ß-Naltrexol in fetal channel. - Co-culture device: 2.5% and 2.2% of initial maternal concentration NTX and 6ß-Naltrexol in fetal channel. - Epithelial and endothelial cell layers disruption after 8 h exposure to NTX/6ß-Naltrexol. - Permeable placental barrier after 6 h exposure to NTX-6ß-Naltrexol.	Pemathilaka et al. [21]
Caffeine	Rate of placental caffeine transport	BeWo cells (Trophoblast cell line) HUVECs (Endothelial cell line)	Substance tested: Caffeine concentration: 1.3 mM Exposure 7.5 h Placental barrier: Flow rate: 50 μL/h Porous membrane: polyethylene terephthalate, pore size: 0.4 μm ECM: Entactin collagen IV-laminin coating (10 μg/mL)	In-house manufactured using soft lithography, PDMS-based	- Steady-state concentration fetal side: 0.0033 mg/mL (6.5–7.5 h). - Steady-state concentration maternal side: 0.1513 mg/mL (6.5–7.5 h). - Cell detachment after 7.5 h causing fluctuations in caffeine concentrations.	Pemathilaka et al. [22]
Glyburide	Active placental drug transport	BeWo b30 cells (Trophoblast cell line) HPVECs (Endothelial cell line)	Substance tested: Heparin size: 3000–15,000 kDa Exposure 5 h Glyburide concentration: 1.0 × 10^−3^ mM (BODIPY-conjugated Glyburide) Exposure 3 h Placental barrier: Flow rate: 100 μL/h Porous membrane: polycarbonate, pore size: 1 μm ECM: Fibronectin coating (0.1 mg/mL)	In-house manufactured using soft lithography, PDMS-based	- Microvilli formation on apical surface of maternal channel. - No syncytialization of the entire monolayer. - Passage of heparin into the fetal compartment was prevented confirming strong integrity of the placental barrier. - BCRP efflux activity limits transfer of glyburide from maternal to fetal compartment.	Blundell et al. [2]
Rosuvastatin/ Pravastatin	Placental drug transport, efficacy and kinetics of statins	BeWo cells (Trophoblast cell line) HUVECs (Endothelial cell line)	Substance tested: Heparin (0.1 mg/mL) Pravastatin concentration: 4.7 × 10^−4^ mM Rosuvastatin concentration: 4.2 × 10^−4^ mM Exposure 4–24 h Placental barrier: Array of 24 microchannels, 5 μm in height, 30 μm in width, 300/600 μm in length, connecting syncytiotrophoblast, cytotrophoblast and endothelial cell compartments ECM: Collagen type I coating (10 μg/mL)	In-house manufactured using soft lithography, PDMS-based	- Heparin did not cross the device after 8 h, with or without cells. - Transport of Rosuvastatin and Pravastatin across the placenta barrier within 8 h in device containing cells. - Pravastatin was metabolized by all cell layers within after 24 h. - Rosuvastatin was metabolized by both trophoblast layers after 8 and 24 h. - Under inflammatory conditions, Pravastatin increases both pro-inflammatory (IL-6) and anti-inflammatory (IL-4, IL-10) cytokines, whereas Rosuvastatin increases only anti-inflammatory cytokines (IL-4).	Richardson et al. [23]

**Table 5:** Key characteristics of included studies on nanoparticles.

Type of Testing	Aim	Cultured Cells	Properties	Chip Type	Main Findings	Reference
Titanium dioxide nanoparticles	Placental responses to nanoparticles	BeWo cells (Trophoblast cell line) HUVECs (Endothelial cell line)	Particles: TiO_2_-nanoparticles: 50 or 200 μg/mL Size: 50 nm (diameter) Exposure 24 h Placental barrier: Flow rate: 20 μL/h Shear stress: 0.03 dyne/cm^2^ ECM barrier: Matrigel ECM: Chitosan coating (2%)	In-house manufactured using soft lithography, PDMS-based	- Fluid shear stress induced microvilli formation in trophoblast cells. - High TiO_2_ particle concentrations stimulated ROS generation. High TiO_2_ particle concentrations induced damage to placental barrier. - Disrupted permeability of the placental barrier upon exposure to TiO_2_ particles (high/low concentrations). - Higher number of macrophages adhered on trophoblast layer after exposure to TiO_2_-nanoparticles, immune cell behaviour impaired upon exposure to nanoparticles.	Yin et al. [24]
Carboxyl-modified polystyrene microparticles (PS-MPs)	Systemic (in)direct embryotoxicity	BeWo b30 cells (Trophoblast cell line) Embryoid bodies	Particles: PS-MPs concentration: 1, 10 or 100 μg/mL Size: 500 nm (diameter) Exposure 72 h Placental barrier: Flow rate: 276 μL/h Shear stress: 0.0003 dyne/cm^2^ Porous membrane: polyethylene terephthalate, pore size: 3 μm ECM: Collagen type I coating (2%)	In-house manufactured using soft lithography, PDMS-based	- Dose-dependent accumulation and aggregation of PS-MPs in vesicle-like structures in trophoblast cells. - Enhanced fraction of dead cells in the placental barrier upon exposure to increased concentration of PS-MP, but no loss of barrier integrity. - No embryotoxic effects were observed upon direct PS-MP exposure. - No PS-MP translocation across the placental barrier. - ATP content of embryoid bodies was reduced after exposure to PS-MPs.	Boos et al. [25]
CSA-conjugated PEGylated liposomal nanocarriers	Trophoblast cell uptake under dynamic conditions and after chemically induced syncytialization	BeWo b30 cells (Trophoblast cell line) HUVECs (Endothelial cell line)	Particles: Liposome concentration: 5 × 10^6^–5 × 10^8^ particles/mL Size: 93.7 nm (hydrodynamic diameter conjugated liposomes) 48.8/49.3 nm (hydrodynamic diameter unconjugated liposomes) Exposure 48/72 h Placental barrier: Flow rate: 1373/4 μL/h Shear stress: 0.025 dyne/cm^2^ ECM: Gelatin coated (concentration not reported)	iBidi μ-Slide I^0.4^	- Under dynamic conditions trophoblasts showed a higher extent of syncytialization and a higher concentration of ß-hCG was secreted. - Significant higher production of ß-hCG was achieved by trophoblasts after being exposed to forskolin treatment for 48 and 72 h. - Increased cell uptake of liposomes was caused by shear stress combined with increasing exposure to forskolin. - Under dynamic conditions the ß-hCG secretion and syncytialization were significantly increased.	Abostait et al. [10]

**Table 6:** Key characteristics of included study under conditions of bacterial infection.

Type of Testing	Aim	Cultured Cells	Properties	Chip Type	Main Findings	Reference
Bacterial infection (*E. coli*)	Placental inflammatory responses with bacterial infection	BeWo cells (Trophoblast cell line) HUVECs (Endothelial cell line)	Particles: *E. coli* (rod-shaped bacterium 0.5 μm in width, 2 μm in length) Exposure 6 h Placental barrier: Flow rate: 10–40 μL/h Porous membrane: polyethylene terephthalate, pore size: 0.4 μm ECM: Collagen type I coating (0.1 mg/mL)	In-house manufactured using soft lithography, PDMS-based	- Microvilli formation in maternal channel by trophoblasts. - High GLUT1 expression in maternal compartment. - *E. coli* causes overproduction of inflammatory cytokines by trophoblasts. - Activation of maternal innate immune system caused by bacterial infection leading to macrophages production. - Secretion of hCG-ß was lowered under hypoxic conditions and even further decreased under ischemic conditions.	Zhu et al. [26]

**Table 7:** Key characteristics of included studies on preeclampsia.

Type of Testing	Aim	Cultured Cells	Properties	Chip Type	Main Findings	Reference
Preeclampsia (TNF-α)	Assess effects inflammatory conditions in preeclampsia	ACH-3P (Trophoblast cell line) HUVECs (Endothelial cell line)	Substance tested: TNF-α concentration: 1.0 × 10^−5^ g/L Exposure 24/72 h Placental barrier: Flow rate: pressure gradient ECM barrier: Collagen type I gel (2.5 mg/mL)	AIM Biotech idenTx chip	- Increased plasma and placental FKBPL and Gal-3 expression in preeclampsia patients. - Increased trophoblast migration in the presence of endothelial cells in the chip model. - Reduced endothelial vascular network formation by trophoblasts. - Increased FKBPL and Gal-3 signalling after trophoblasts and endothelial cells were treated with TNF-α - TNF-α treatment (72 h) impaired vascular network formation.	Ghorbanpour et al. [30]
Cell invasion	Trophoblast invasion with intraluminal flow	HTR8/SVneo cells (Trophoblast cell line) HUVECs (Endothelial cell line)	Substance tested: Folic acid concentration: 2.3 × 10^−4^ mM Exposure 24/48/72 h Placental barrier: Flow rate: 0.6, 3, 6 and 60 μL/h Shear stress: 0.046, 0.228, and 0.457 dyne/cm^2^ Barrier pillar spacing: 3 μm ECM: Fibronectin coating (200 μg/mL)	SynVivo 3D microfluidic chip	- The permeability of the endothelial barrier increases with faster flow speed (60 μL/h). - Invasion of HTR8/SVneo cells was observed upon exposure to folic acid in a co-cultured model with HUVECs. - Enhanced HTR8/SVneo cells invasiveness upon exposure to folic acid in the absence of HUVECs.	Pu et al. [31]
Oxygen levels	Effect of trophoblast invasion through oxygen level control	HTR8/Svneo (Trophoblast cell line) HUVECs (Endothelial cell line)	Conditions tested: Normoxic condition: 21% O_2_ Hypoxic conditions: 3% O_2_ Exposure 12/24/26 h Placental barrier: Flow rate: static system ECM barrier: Matrigel ECM: Fibronectin coating (40 μg/mL)	In-house manufactured using soft lithography, PDMS-based	- Under hypoxic conditions HTR8/SVneo cell invasion as well as traveled distance increased with exposure time. - Under normoxic conditions no increase in invasion distance of HTR8/SVneo cells was observed with longer exposure times. - Decrease in MMP-2 expression after 36 h exposure in hypoxic and normoxic environment. - Decrease in MMP-9 expression after 12–36 h exposure in normoxic environment. - Increase in MMP-9 expression after 36 h exposure in hypoxic environment.	Cho et al. [28]
Oxygen levels	Effect of oxygen level on trophoblast migration	HTR8/SVneo cells (Trophoblast cell line) HUVECs (Endothelial cell line)	Conditions tested: Normoxic condition: 21% O_2_ Hypoxic conditions: 3% O_2_ Exposure 6/8 days Placental barrier: Flow rate: static system ECM barrier: Gelatin-methacrylate (GelMA) ECM: Fibronectin coating (50 ng/mL)	In-house manufactured using soft lithography, PDMS-based	- In hypoxic environment higher expression of MMP-9 and MMP-2 mRNA was observed in trophoblast cells. - Trophoblast migration was promoted in GelMA structure by hypoxia.	Ko et al. [27]
Oxygen levels	Understand the underlying mechanism of preeclampsia	BeWo b30 cells (Trophoblast cell line) HUVECs (Endothelial cell line)	Conditions tested: Normoxic condition: 20% O_2_ Hypoxic conditions: 1% O_2_ Exposure 24/48/72 h Placental barrier: Flow rate: static system ECM barrier: Collagen-I/collagen-IV mixture (3:1 ratio)	3-lane Organo Plate (MIMETAS)	- Multi-drug resistance protein (MRP) and breast cancer resistance protein (BCRP) transporter activity was observed in placental barrier. - Normoxic and hypoxic environment did not affect syncytium permeability. - Decreased syncytium permeability was observed under ischemic conditions. - Complete loss of microvilli in ischemic environment. - Expression of glucose transporter-1 (GLUT1) was decreased by 25% under ischemic conditions.	Rabussier et al. [29]

## Data Availability

As no original data are used, we are not able to share data.
